# Additional radiation boost to whole brain radiation therapy may improve the survival of patients with brain metastases in small cell lung cancer

**DOI:** 10.1186/s13014-018-1198-4

**Published:** 2018-12-18

**Authors:** Han Sun, Liming Xu, Youyou Wang, Junhua Zhao, Kunpeng Xu, Jing Qi, Zhiyong Yuan, Lujun Zhao, Ping Wang

**Affiliations:** Department of Radiation Oncology, Tianjin Medical University Cancer Institute and Hospital, National Clinical Research Center for Cancer, Key Laboratory of Cancer Prevention and Therapy, Tianjin, Tianjin’s Clinical Research Center for Cancer, Tianjin, 300060 China

**Keywords:** Small cell lung cancer, Brain metastasis, Whole brain radiation therapy, Dose escalation, Prognosis, Overall survival

## Abstract

**Background:**

The role of the dose escalation strategy in brain radiotherapy for small cell lung cancer (SCLC) patients with brain metastases (BMs) has not been identified. This study aims to determine whether an additional radiation boost to whole brain radiation therapy (WBRT) has beneficial effects on overall survival (OS) compared with WBRT-alone.

**Methods:**

A total of 82 SCLC patients who were found to have BMs treated with WBRT plus a radiation boost (*n* = 33) or WBRT-alone (*n* = 49) from January 2008 to December 2015 were retrospectively analyzed. All patients were limited-stage (LS) SCLC at the time of the initial diagnosis, and none of them had extracranial metastases prior to detection of BMs. The primary end point was OS.

**Results:**

The median OS for all of the patients was 9.6 months and the 6-, 12- and 24-months OS rates were 69.1, 42.2 and 12.8%, respectively. At baseline, the proportion of more than 3 BMs was significantly higher in the WBRT group than in the WBRT plus boost group (*p* = 0.0001). WBRT plus a radiation boost was significantly associated with improved OS in these patients when compared with WBRT-alone (13.4 vs. 8.5 months; *p* = 0.004). Further, the survival benefit still remained significant in WBRT plus boost group among patients with 1 to 3 BMs (13.4 vs. 9.6 months; *p* = 0.022).

**Conclusion:**

Compared with WBRT-alone, the use of WBRT plus a radiation boost may prolong survival in SCLC patients with BMs. The dose escalation strategy in brain radiotherapy for selected BMs patients with SCLC should be considered.

## Background

Small cell lung cancer (SCLC), which is well known for its aggressiveness and early dissemination, is associated with a high propensity for brain metastases (BMs) [1]. According to statistics, 10% of SCLC patients have BMs at the time of the initial diagnosis, which increases to 50% of patients by 2 years after the diagnosis [2]. For those SCLC patients with BMs, the prognosis of the disease is generally poor with a median survival of fewer than five months [3]. Based on the 2018 National Comprehensive Cancer Network (NCCN) guidelines, whole brain radiation therapy (WBRT) is the standard treatment for SCLC patients with BMs [4], and 50–80% of patients respond positively to treatment [3].

MRI is extensively utilized to detect BMs in the clinic and more microscopic tumor infiltrates can be discovered during the early stages of metastasis. The detection of BMs has increased by more than 14% since the beginning of the MRI era [5]. Patients with a limited number (1 to 3) of BMs are more readily diagnosed, which has led to favorable patient outcomes and increased life expectancies [6]. With the current expansion of the highly effective stereotactic surgery (SRS) as a locally ablative treatment modality, randomized trials have demonstrated that BMs could be safely and effectively treated with SRS-alone in patients having up to 10 BMs [7, 8]. While some retrospective studies have recommended SRS as the first-line treatment for providing effective local control of BMs in SCLC [9, 10], SRS-alone is less frequently used in this cohort because of the development of diffuse intracranial metastases. Previous randomized trials showed that the combination of SRS and WBRT could improve the overall survival of cancer patients with a single BM [11, 12]. However, the dose escalation strategy has not been extensively investigated in SCLC patients, and it is unknown whether the combination of WBRT with an additional radiation boost is a feasible treatment option to increase the survival of SCLC patients with BMs. In this study, we retrospectively evaluate the outcome and prognosis of SCLC patients with BMs when treated with WBRT plus a radiation boost or WBRT-alone.

## Methods

### Patient selection and review of medical records

This is a single institutional retrospective study that was approved by the local institutional review board. Eligible subjects included patients who had cytologically- or histologically-proven limited-stage (LS) SCLC according to the classification of the Veterans’ Affairs Administration Lung Cancer Study Group (VALG) at the time of the initial diagnosis and had undergone WBRT-alone or WBRT plus a boost of radiation after confirmation of BMs. Those patients who had extracranial metastases or were treated with prophylactic cranial irradiation (PCI) prior to the detection of BMs were excluded from this study. In total, 82 patients in our hospital from January 2008 to December 2015 were included. BMs were identified with gadolinium-enhanced MRI in 78 patients, while computed tomography (CT) scans were used to detect BMs in the other four patients. Brain MRI surveillance was performed every 3 months during the first 2 years, then every 6 months for 3 years. Lack of extracranial metastases prior to the discovery of BMs was confirmed by CT of the chest and abdomen. The patients’ clinical, treatment, and demographic characteristics were recorded, including gender, age, smoking history, weight, Karnofsky performance status (KPS), number of BMs, maximum diameter of the largest tumor, treatment regimen before or after detection of BMs, extracranial disease status, date of diagnosis of BMs, and date of death or final follow-up visit. Details of patients’ characteristics are provided in Table 1. Progressive extracranial disease was defined as uncontrolled primary tumor or presence of extracranial metastases.

**Table 1:** patients’ clinical and treatment characteristics and survival-related factors on OS in univariate analysis

Characteristics	No. of patients (%)	Median OS (months)	*P* value
Sex			
Male	64 (87.0)	9.3	0.860
Female	18 (22.0)	10.3	
Age (y)			
Median	59		
Range	39–73		
< 65	44 (53.7)	12.3	0.349
≥ 65	38 (46.3)	9.2	
KPS			
≥ 70	76 (92.7)	11.2	0.032^*^
> 70	6 (7.3)	5.0	
Smoking history			
Yes	64 (78.0)	9.2	0.647
No	18 (22.0)	10.3	
Weight loss≥5%			
Yes	26 (31.7)	9.3	0.734
No	56 (68.3)	10.2	
Number of BMs			
1	21 (25.6)	12.6	0.034^*^
2–3	24 (29.3)	10.3	
> 3	37 (45.1)	6.8	
Interval from diagnosis of SCLC to BMs (month)			
≤ 10	40 (48.7)	9.2	0.204
> 10	42 (51.3)	10.3	
Maximum diameter of the largest tumor (cm)			
≤ 2.0	44 (53.6)	12.6	0.002^*^
> 2.0	38 (46.3)	9.4	
Symptomatic BMs			
Yes	27 (32.9)	9.0	0.039^*^
No	55 (67.1)	10.3	
Treatment regimen before BMs			
Concurrent CRT	28 (33.7)	9.8	0.880
Sequential CRT	54 (66.3)	9.6	
Extracranial disease status			
Progressive	15 (18.3)	5.1	0.016^*^
Control	67 (81.7)	10.3	
Brain RT regimen			
WBRT	49 (59.8)	8.5	0.004^*^
WBRT plus boost	33 (40.2)	13.4	

*p < 0.05 was considered significant. *KPS* Karnofsky performance score, *BM* brain metastases, *CRT* chemoradiotherapy, *RT* radiation therapy, *WBRT* whole brain radiotherapy

### Treatment strategy

All patients received sequential or concurrent chemoradiotherapy after confirming the initial SCLC diagnosis. The treatment regimens were platinum-based doublet chemotherapy with cisplatin (30 mg/m [2] from days 1 to 3) or carboplatin (500 mg for day 1) combined with etoposide (100 mg from days 1 to 5). For patients receiving sequential or concurrent chemoradiotherapy, the median number of chemotherapy cycles was 4 with a range of 2 to 6. Thoracic radiotherapy was delivered by 6 MV linear accelerators. In addition, 77 patients received intensity-modulated radiation therapy (IMRT) and five of them received 3D conformal radiotherapy (3D-CRT). For all patients, the tumor and metastatic lymph nodes were defined as the gross tumor volume (GTV). The clinical target volume (CTV) encompassed the tumor bed and the draining area of metastatic lymph nodes before chemotherapy, which was expanded from the GTV by a 5 mm uniform margin. The planning target volume (PTV) was outlined with a 5–10 mm margin to the CTV. The radiation dose was 50–63 Gy in 25–30 fractions, 1.8–2.1 Gy per fraction at one fraction per day.

After confirming the BMs, 49 patients received WBRT-alone, while the other 33 patients underwent WBRT plus a radiation boost. WBRT was performed with 6 MV photon beams using opposed lateral fields (90° and 270°) with a total dose of 30–36 Gy (3 Gy per fraction administered in 10–12 fractions at one fraction per day). The additional radiation boost was administered using a Cyberknife (Accuracy, Sunnyvale, California, USA) after the WBRT in 20 patients. The GTV encompassed contrast-enhancing tumor on MRI and were reviewed by the radiation oncologist and the neurosurgeon based on the tumor volume, tumor location, and neurological symptoms. The PTV was defined as the 1 to 2 mm margin to the GTV. The administered radiation dose was 8.5–19 Gy in 1–3 fractions with 6.3–18.0 Gy per fraction and one fraction per day. The IMRT simultaneous integrated boost WBRT (IMRT-SIB-WBRT) was administered in 13 patients. The GTV was contoured based on the tumor from contrast-enhanced MRI scans. The PTV of metastases (PTV_m_) was defined as the 3 mm margin to the GTV with the dose of 35–50 Gy in 10 fractions with 3.5–5 Gy per fraction and one fraction per day. In general, we treated BMs less than 10 mm in maximum diameter with a prescription of 50 Gy; BMs larger than 10 mm but smaller than 30 mm with 40 Gy; and BMs larger than 30 mm and less than 40 mm with 35Gy. The prescription of dose fractionation was consistent with previous clinical trials [13, 14]. The PTV was expanded from the contour of the brain by the 3 mm uniform margin with the dose of 30 Gy in 10 fractions with 3 Gy per fraction and one fraction per day.

### Data analysis and statistical considerations

As the primary endpoint for this study, the overall survival (OS) was defined as the time from the date of BM diagnosis to death or the final follow-up visit. The follow-up schedule began with the time of treatment and the final follow-up time on May 1, 2017. The date of death or the final follow-up visits were obtained from hospital records or correspondence with the referring physician or the family of patients directly. The Kaplan-Meier method was used to compute the OS of patients in different groups. The log-rank test was used to compare the survival curves and assess the significance. The comparison of clinical characteristics was performed using the chi-square test. The multivariate analysis was conducted using the Cox proportional hazards model to examine factors associated with increased hazard of death. All *p*-values were two-sided, with *p* < 0.05 being considered statistically significant. SPSS 18.0 software (IBM, Chicago, IL, USA) was applied to perform the statistical analyses.

## Results

### Overall survival

**Fig. 1:**
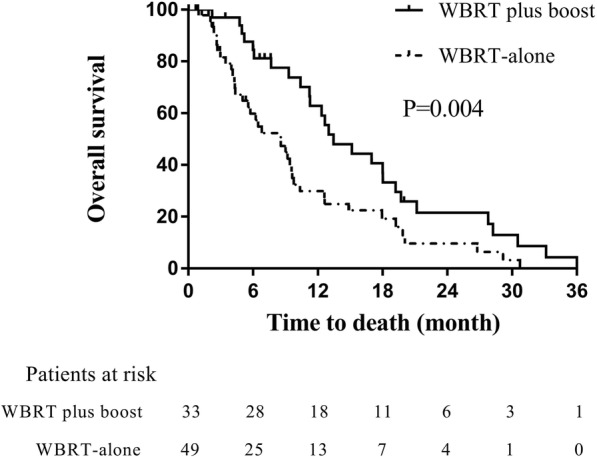
Comparison of overall survival between WBRT plus boost and WBRT-alone in all patients

For all of the patients, the median follow-up visit was at 11.4 months (range, 0.3–95.4). The median OS was 9.6 months and the 6-, 12-, and 24-month OS rates were 69.1, 42.2, and 12.8%, respectively. At baseline, the proportion of more than 3 BMs was significantly higher in the WBRT group than that in the WBRT plus boost group (*p* = 0.0001), which would be advantageous for the WBRT plus boost group (Tab. 2). The median OS in the WBRT group (*n* = 49) and the WBRT plus boost group (*n* = 33) were 8.5 months and 13.4 months, respectively. The 6-, 12-, and 24-month OS rates in the WBRT group were 59.8, 29.9, 9.6%, respectively, versus 84.5, 62.7, and 21.5% in the WBRT plus boost group, respectively (*p* = 0.004; Fig. 1).

**Table 2:** Distribution of the patient treatment and clinical characteristics in two treatment groups

Characteristics	WBRT (%)	WBRT plus boost (%)	P value
Sex (male)	41 (81.7)	23 (69.7)	0.134
Age (median)	59	61	0.572
KPS ≥ 70	43 (87.8)	33 (100)	0.057
Smoking history(yes)	37 (75.5)	27 (81.8)	0.499
Weight loss>5%	18 (36.7)	8 (24.2)	0.333
Number of BM			
1	5 (10.2)	16 (48.5)	0.0001^*^
2–3	13 (26.5)	11 (33.3)	
> 3	31 (63.3)	6 (18.2)	
Interval from diagnosis of SCLC to BMs (< 10 month)	21 (42.9)	18 (56.2)	0.238
Maximum diameter of the largest tumor (≤2.0 cm)	23 (46.9)	17 (51.5)	0.213
Treatment regimen before BM (Sequential CRT)	36 (73.5)	18 (54.5)	0.076
Symptomatic BM	18 (36.7)	9 (27.3)	0.371
Progressive extracranial diseases	11 (22.4)	4 (12.1)	0.236

*p < 0.05 was considered significant. *KPS* Karnofsky performance score, *BM* brain metastases, *CRT* chemoradiotherapy, *RT* radiation therapy, *WBRT* whole brain radiotherapy

**Fig. 2:**
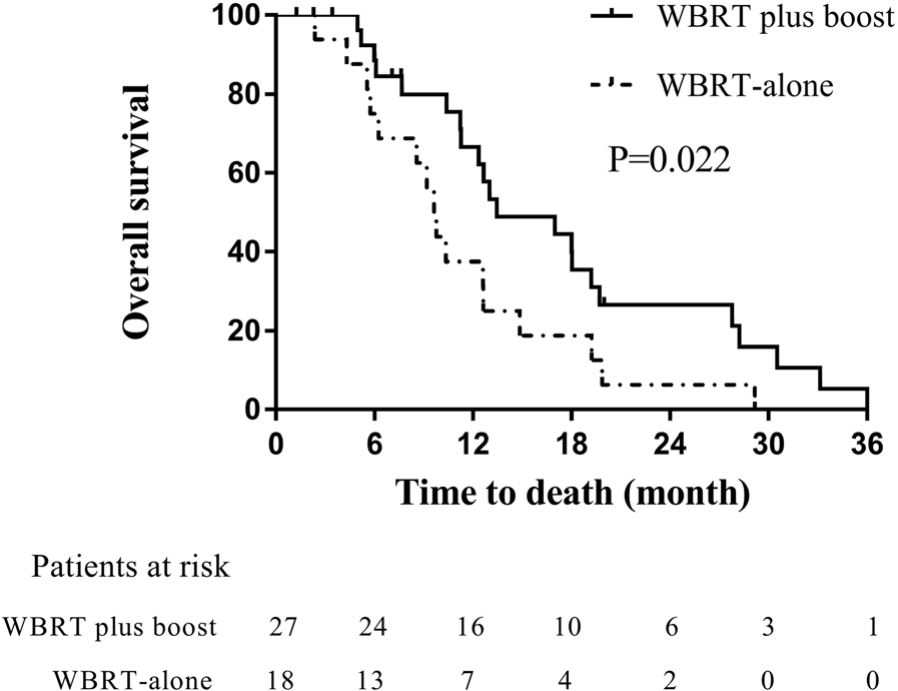
Comparison of overall survival between WBRT plus boost and WBRT-alone in patients with 1 to 3 BMs

To minimize the difference in the number of BMs, we analyzed those patients with 1 to 3 BMs. The median OS for the WBRT group (*n* = 18) and the WBRT plus boost group (*n* = 27) were 9.6 months and 13.4 months, respectively. The 6-, 12- and 24-month OS rates in the WBRT group were 75.0, 37.5, 6.3%, respectively, versus 84.4, 66.7, and 26.6% in the WBRT plus boost group, respectively. The survival benefit in the WBRT plus boost group remained significant (*p* = 0.022; Fig. 2).

### Factors associated with the overall survival univariate analysis

In the univariate analysis, a limited number (1 to 3) of BMs (*p* = 0.034), KPS ≥ 70 (*p* = 0.032), asymptomatic BMs (*p* = 0.039), controlled extracranial diseases (*p* = 0.016), and maximum diameter of the largest tumor≤2.0 cm (*p* = 0.002) were significantly associated with increased survival (Tab. 1). No significant differences were observed in age, sex, weight loss, smoking history, treatment regimen before BMs, or the interval from diagnose of SCLC to BMs (*p* > 0.204 for all factors).

**Fig. 3:**
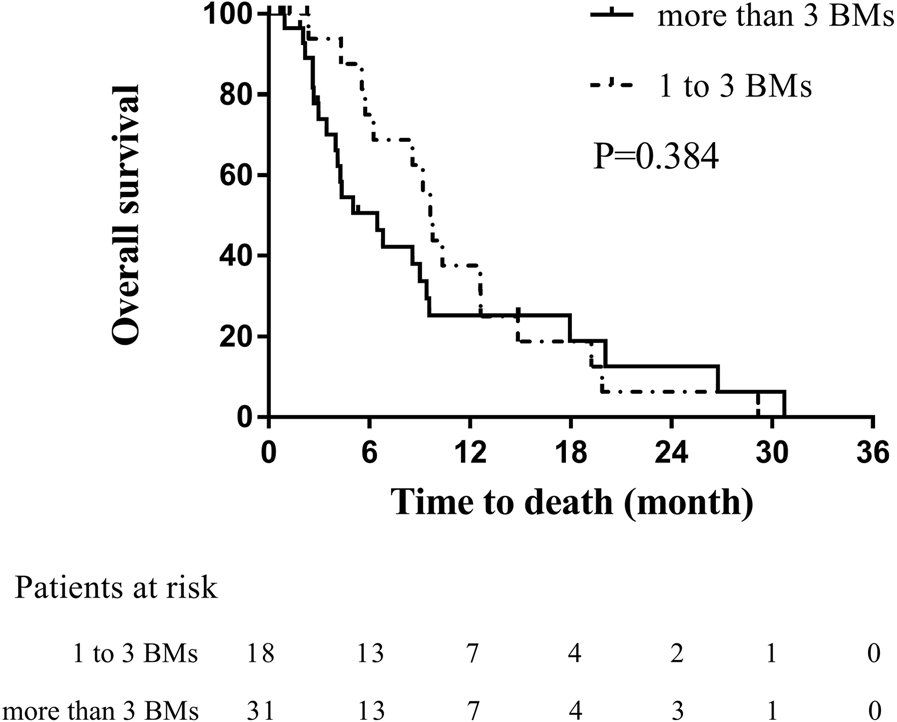
Comparison of overall survival between patients with 1 to 3 BMs and more than 3 BMs in WBRT-alone group

In the WBRT group, 18 patients had 1 to 3 BMs, and 31 patients had more than 3 BMs. There was no significant difference in OS among the groups (*p* = 0.384). The median, 6-, 12-, and 24-month survival rates were 9.6 months, 75.0, 37.5 and 6.3% for patients who had 1 to 3 BMs and 6.4 months,50.6, 25.3, and 12.7% for patients with more than 3 BMs (Fig. 3).

### Factors associated with the overall survival multivariate analysis

The covariates with *p*-values of less than 0.05 from univariate analysis were further analyzed in the multivariate Cox proportional hazards analysis. Therefore, number of BMs, KPS, symptomatic BMs (no vs. yes), extracranial disease status (controlled vs. progressive), maximum diameter of the largest tumor, and WBRT-alone vs. WBRT plus boost were introduced into the Cox regression model (Tab. 3). WBRT plus boost (*p* = 0.031), controlled extracranial disease (*p* = 0.016), smaller tumor size (*p* = 0.015) and asymptomatic BMs (*p* = 0.005) retained significance for improving OS in the multivariate analysis. However, the difference was not significant in KPS and the number of BMs (*p* > 0.535 for all factors).

**Table 3:** Survival-related factors on OS in multivariate analysis

Characteristics	HR of death (95% CI)	P value
WBRT vs. WBRT plus boost	0.681(0.473–0.958)	0.031^*^
Number of BM	1.034(0.719–1.288)	0.795
KPS	1.014(0.970–1.061)	0.535
Symptomatic BM (no vs. yes)	2.433(1.302–4.546)	0.005^*^
Maximum diameter of the largest tumor	1.420(1.124–1.794)	0.015^*^
Extracranial disease (control vs. progression)	2.234(1.158–4.310)	0.016^*^

*p < 0.05 was considered significant. *WBRT* whole brain radiotherapy, *BM* brain metastases, *KPS* Karnofsky performance score, *RT* radiation therapy

## Discussion

This single-institution study retrospectively evaluated a cohort of LS-SCLC patients who had not suffered from extracranial metastases before being diagnosed with BMs. Our results demonstrated that WBRT plus a radiation boost was significantly associated with improved OS in these patients when compared with WBRT-alone.

It is generally believed that SCLC-derived BMs are rarely solitary and characterized by early intracranial dissemination [1].Even in stage I-III SCLC patients treated with surgical resection, the cumulative incidence of BMs is up to 25% [15]. WBRT remains the standard treatment because of the limited life expectancy of SCLC patients with BMs, in addition to the lack of other effective treatments available [16]. Considering the high frequency of intracranial recurrences, SRS or surgery-alone are rarely curative in SCLC patients. However, there is a growing body of evidence suggesting feasibility of SRS alone for treatment of BMs in patients with SCLC. Currently, a German phase 2 study is evaluating this concept [17] and the optimal management of BMs remains obscure. Several clinical trials have reported the use of WBRT plus a radiation boost in the management of BMs. Andrews et al. [11] recruited 331 patients with 1 to 3 BMs. In the entire cohort, WBRT plus SRS significantly improved the local control of intracranial metastases at 1 year (82% vs. 71%, *p* = 0.013), as well as the KPS scores (43% vs 27%, *p* = 0.03), compared with WBRT-alone. In addition, a survival benefit was observed in patients with a single BM with the median OS of 6.5 and 4.9 months for WBRT and WBRT plus SRS, respectively (*p* = 0.039). Of note, only 24 patients (7%) in this cohort were diagnosed with SCLC. A secondary analysis of JROSG 99–1 compared WBRT plus SRS with SRS-alone in 132 non-small cell lung cancer (NSCLC) patients who had 1 to 4 BMs. Aoyama et al. [12] reported that WBRT plus SRS significantly improved the OS in the subgroup of DS-GPA 2.5–4.0 with a median OS of 16.7 months vs. 10.6 months, respectively (*p* = 0.04).

Is WBRT plus a radiation boost also a feasible treatment for patients with SCLC? Currently, no previous studies have comprehensively investigated this issue. Wegner et al. [9] compared 44 SCLC patients who underwent SRS with or without WBRT, and WBRT plus SRS was found to be associated with improved OS in patients from 6 months to 14 months after treatment (*p* = 0.04). However, this result should be interpreted cautiously as the sample size was small, consisting of only six patients who were treated in the WBRT plus SRS group. In a large-scale cohort of 4259 patients, Sperduto et al. [6] reviewed the records of 299 SCLC patients with BMs. From the cohort, 247 patients were treated with WBRT-alone, while 21 patients were treated with WBRT plus SRS. The OS was significantly higher in the WBRT plus SRS patients with the median OS of 15.23 months vs. 3.87 months, respectively (*p* = 0.003). Traditionally, WBRT patients may have more BMs and worse functional autonomy (KPS) than patients treated with WBRT plus SRS. However, Sperduto et al. failed to correct the differences between two treatment groups. Our study validated these characteristics and further analyzed those patients with 1 to 3 BMs. The OS benefit in the WBRT plus boost group remained significant, while the median OS in the WBRT plus boost group was similar to that from previous studies [6, 9].

In several previous analyses, some prognostic factors such as KPS, age, extracranial metastases status, number of BMs, and metachronous disease have been identified in SCLC patients with BMs [6, 18–20]. In this study, symptomatic BMs, extracranial disease status and maximum diameter of the largest tumor were significantly associated with OS in both the univariate and the multivariate analyses. Furthermore, the OS was significantly affected by the number of BMs and KPS in the univariate analysis.

One of the novel discoveries of this study was that there was no significant difference in OS between patients with 1 to 3 BMs and more than 3 BMs in the WBRT group (*p* = 0.384). Based on the DS-GPA classification, the number of BMs was a significant prognostic factor [6]. However, the patients in this cohort were managed using different treatment modalities, including WBRT, SRS, or WBRT plus SRS or surgery, which may lead to inappropriate conclusions. Bernhardt et al. [19] retrospectively analyzed 229 SCLC patients who were treated with WBRT, and showed that the number of BMs was not a significant prognostic factor in the univariate (*p* = 0.06) or the multivariate analysis (*p* = 0.511). Another study compared the different courses of WBRT in 146 SCLC patients and showed that the number of BMs was significantly associated with improved OS (*p* = 0.011) and local intracranial control (*p* = 0.027) [21]. These contradictory results may be due, at least in part, to the different size of BMs. For this reason, we further analyze the tumor size in the entire cohort. Similar to previous studies [7], the patients with the smaller tumor size were significantly associated with improved OS in both the univariate (*p* = 0.002) and the multivariate analyses (*p* = 0.015). While WBRT-alone could provide active remission for small or subclinical lesions, it might have limited effect against larger metastases.

There are several limitations to this analysis. First, the proportion of patients with multiple BMs was significantly higher in the WBRT group. Although we further analyzed the patients 1 to 3 BMs, the results may be limited by the treatment selection bias. Secondly, because of missing data points about intracranial recurrence and uniform toxicity assessments, we failed to evaluate local intracranial control and dose escalation-related toxicities in the present study. Thirdly, this is a small-sample retrospective study with certain inherent bias, and the conclusion should be validated in further prospective studies.

## Conclusions

To our knowledge, this is the first retrospective study that evaluates a dose escalation strategy in the management of BMs in SCLC patients comprehensively. WBRT plus a radiation boost delivered with stereotactic radiotherapy or a simultaneous integrated boost, may prolong the OS of patients when compared with WBRT-alone in SCLC patients with BMs. The dose escalation strategy in brain radiotherapy for selected BMs patients with SCLC should be taken into consideration. The local intracranial control and the dose escalation-related toxicities for patients treated in WBRT plus boost need to be evaluated in further studies.

## Abbreviations

3D-CRT: 3D conformal radiotherapy
BMs: Brain metastases
CT: Computed tomography
CTV: Clinical target volume
GTV: Gross tumor volume
IMRT: Intensity-modulated radiation therapy
IMRT-SIB-WBRT: Intensity-modulated radiation therapy simultaneous integrated boost whole brain radiation therapy
KPS: Karnofsky performance status
LS-SCLC: Limited-stage small cell lung cancer
NCCN: National Comprehensive Cancer Network
OS: Overall survival
PCI: Prophylactic cranial irradiation
PTV: Planning target volume
PTV_m_: Planning target volume of metastases
SCLC: Small cell lung cancer
SRS: Stereotactic surgery
WBRT: Whole brain radiation therapy
